# The mental health of fly-in fly-out workers before and during COVID-19: a comparison study

**DOI:** 10.1080/00049530.2023.2170280

**Published:** 2023-02-19

**Authors:** Jessica M. Gilbert, Laura S. Fruhen, Cindy T. Burton, Sharon K. Parker

**Affiliations:** a Centre for Transformative Work Design, Future of Work Institute, Curtin University, Perth, Australia; b School of Psychological Science, Psychology at Work Lab, University of Western Australia, Perth, Australia

**Keywords:** Remote work, wellbeing, burnout, psychological distress, FIFO

## Abstract

**Objectives:**

This study gives an overview of the impact of FIFO work on workers’ mental health before and during COVID-19, using three comparison samples as well as norm data. It provides a timely update on FIFO workers' mental health and how it has been impacted during COVID-19.

**Method:**

Comparisons are conducted with three participant samples, namely two FIFO worker samples (one before and one during the Covid pandemic) and a purposefully sampled benchmark sample, and Australian population norm data on mental health. Constructs included in surveys were psychological distress, burnout, suicide intention, as well as social, psychological, and emotional wellbeing.

**Results:**

FIFO workers were found to have worse mental health than the matched benchmark sample, and the Australian norm samples pre-COVID-19. Differences between FIFO workers and the matched benchmark sample persisted for psychological distress and burnout after controlling for demographic factors. Mental ill-health and poor well-being were higher during the COVID-19 pandemic than before.

**Conclusions:**

FIFO workers need to be considered an at-risk group for adverse mental health outcomes, and this is even more so the case during COVID-19. Findings are attributable to the experience of FIFO work as well as the demographic character of the workforce.

## Key Points

What is already known about this topic:

(1) Research findings on FIFO workers' mental health are mixed.

(2) A comprehensive comparison of FIFO worker mental health with the wider Australian population on a range of mental health indicators is needed to provide clarity on this issue.

(3) Impacts of COVID-19 on FIFO worker mental health have been anecdotally reported but have to date not been empirically tested.

What this topic adds:

(1) This study shows that FIFO workers had worse mental health compared to non-FIFO workers before COVID-19 in 2018.

(2) It documents differences in FIFO workers’ mental health before (2018) and during Covid (2020).

(3) The study’s findings clearly identify FIFO workers as an at-risk group for mental health.

The mental health and wellbeing of fly-in fly-out workers (FIFO workers) in Australia is a topic of concern amongst researchers, practitioners, policymakers, and the broader community (Parker et al., 2018). FIFO workplaces involve “work in relatively remote locations where food and lodging accommodation is provided for workers at the work site, but not for their families. Schedules are established whereby employees spend a fixed number of days working at the site, followed by a fixed number of days at home” (Storey, 2001, p. 135). Multiple government inquiries have concluded that FIFO workers operate in a unique environment and are an at risk group for higher levels of psychological distress, loneliness, and suicide risk (Education and Health Standing Committee, 2015; Infrastructure Planning and Natural Resources Committee, 2015). Yet these inquiries and research report that findings are mixed, and more research is needed to provide clarity around the state of FIFO workers' mental health to guide practical intervention (Fruhen et al., 2022). In addition, FIFO work has also undergone some key challenges during COVID-19 with travel restrictions, quarantine needs and border closures having impacted workers and their usual fluctuations between site and home (Gilbert et al., 2020). These challenges’ impact on FIFO workers' mental health is yet to be investigated. To that end, this study provides a timely update on FIFO workers' mental health before and during COVID-19.

Across academic studies, findings are mixed regarding FIFO workers’ mental health relative to other workers (Fruhen et al., 2022). Out of studies that directly compare the mental health and wellbeing of FIFO workers with workers in other forms of employment, many have shown that the mental health and wellbeing of FIFO workers is worse relative to others (e.g., Bowers et al., 2018; Considine et al., 2017; Henry et al., 2013; Lester et al., 2015; Sellenger & Oosthuizen, 2017). Other studies identify no difference between these groups’ mental health (Bradbury, 2011; Clifford, 2009). Further, comparisons with those in other non-mining forms of employment, or in a mining job but living residentially show that FIFO work is associated with better mental health (Bradbury, 2011; Joyce et al., 2013; Miller et al., 2019; Velander et al., 2010). These mixed results have been attributed to the varying quality in the research designs of studies in this area, in particular, the lack of research that uses a matched benchmark sample on key demographics or similar occupations (Parker et al., 2018). Further, a commonly identified confounding factor related to the research findings on FIFO worker mental health is the demographic makeup of this workforce (i.e., male, particular age group, educational background; Considine et al., 2017; Parker et al., 2018). This possible confounding effect highlights the need for research that controls for these attributes or matches samples to discern the impact of FIFO work on worker mental health independent of demographic characteristics. In addition, research that captures the full spectrum of FIFO workers' mental health is lacking, with many studies focusing on one or a couple of mental health aspects. Mental health is not merely the absence of mental illness but rather a state of wellbeing (World Health Organisation, 2013) and as such requires the absence of negative indicators of mental health and presence of positive indicators. Accordingly, in this study, we consider negative mental health indicators (i.e., psychological distress and suicidal thoughts) as well as positive ones (i.e., social, emotional, and psychological wellbeing) to capture FIFO workers' mental health more holistically. In short, a representative and contextualised study providing a comprehensive analysis of FIFO worker mental health that gives more definitive answers regarding the state of FIFO workers’ mental health and wellbeing is needed. Without such a study, it is unclear to what extent FIFO workers’ mental health warrants attention and initiatives towards addressing this issue are hampered by the long-standing debate around the mixed findings.

Further, research from around the globe has identified that COVID-19, while predominantly a physical health crisis, has had a significant impact on mental health (e.g., Biddle et al., 2020). As has been the case for many others, the working conditions of FIFO workers changed significantly in response to the public health measures that needed to be implemented (Gilbert et al., 2020). Of note, for FIFO workers these measures meant often increased social isolation on site, less favourable rosters, and extended periods away from family for many (Gilbert et al., 2020; Trinca, 2020). Concerningly, the resulting changes in response to COVID-19 affected issues that have previously been documented as being connected with worse mental health outcomes in FIFO workers, such as longer on site rosters, inability to visit family during time off, or lack of opportunity to socialise with others on site (Albrecht & Anglim, 2018; Dorow & Jean, 2021; Gardner et al., 2018; Parker et al., 2018). Consequently, the COVID-19 public health measures, while necessary and designed to protect workers, may have had unintended negative side effects for worker mental health and these effects may persist beyond these measures being in place. Establishing the extent to which FIFO workers' mental health may have been affected by these measures is key. Political decision-makers are debating how living with COVID-19 can be managed (Karp, 2021) understanding how the measures put in place may affect FIFO workers can support informed trade-off decision-making and means mental health can be strategically considered. These findings can help understand what FIFO workers have been through, can inform actions and levels of support needed during the return to a new normal, and can shape responses to future crises. Thus, research into the impact of COVID-19 on FIFO workers, is therefore important and, to our knowledge, is lacking in the literature.

To address the issues outlined above, the present study aims to generate insights into the state of FIFO workers’ mental health before and during COVID-19. The present study considers not just mental ill-health, but also positive mental health and wellbeing outcome indicators (in line with Greenspoon & Saklofske, 2001; World Health Organisation, 2013) and controls for key demographic variables where feasible to determine the role of these demographics for FIFO worker mental health. In what follows, we present the results of a series of comparisons, via which the study provides comprehensive insights into FIFO workers’ mental health before and during the COVID-19 pandemic.

## Method

### Participants

Data from three samples were collected for this study, namely a FIFO worker sample in 2018, a purposefully sampled benchmark sample for comparison in 2018, and a further FIFO sample in 2020 (the first wave of COVID-19; see Table 1 for an overview of all samples). In addition to the collected data, data on psychological distress (K10) of the wider Australian population is used from the National Health Survey 2017–2018 in comparisons (Australian Bureau of Statistics, 2018b).

**Table 1.** Overview of demographic characteristics.

Characteristic	Sample		
	FIFO worker 2018 sample (*N* = 3108)	Benchmark 2018 sample (*N* = 326)	FIFO worker 2020 COVID-19 sample (*N* = 362)
Gender			
Male	82.8%	77.3%	81.6%
Female	17.1%	22.7%	18.4%
Other	0.1%	0%	0%
Age			
<24	3.3%	0.6%	2.0%
25–34	29.7%	9.4%	18.3%
35–44	29.4%	20.8%	25.2%
45–54	25.2%	28.9%	22.6%
55+	12.4%	40.3%	31.9%
* M(SD)*	*40.85 (10.59)*	*50.17 (11.31)*	*43.79 (10.82)*
Aboriginal/Torres Strait Islander			
Yes	2.9%	0%	2.9%
No	94.2%	98.4%	90.9%
Prefer not to say	2.9%	1.6%	6.1%
Marital status			
Single, never married	15.6%	13.9%	14.6%
Married/domestic partnership	74.6%	71.9%	75.1%
Widowed, divorced, separated	9.8%	14.2%	10.4%
Children			
0	39.1%	38.7%	40.8%
1	13.0%	12.3%	16.5%
2	27.3%	30.6%	23.6%
3	13.4%	12.3%	12.0%
4	4.2%	3.9%	5.5%
5	1.4%	1.9%	0.3%
6 or more	1.4%	0.3%	1.3%
Age youngest child			
0–2 months	8.3%	3.2%	4.9%
1 up to 3 years	15.7%	7.9%	13.7%
3 up to 5 years	13.1%	4.7%	13.1%
6 up to 8 years	8.8%	7.9%	8.2%
8 up to 2 years	13.7%	12.1%	9.8%
12 up to 8 years	16.0%	17.9%	18.6%
Over 18	24.3%	46.3%	31.7%
Highest level of education			
Primary school	0.2%	0.0%	0.0%
Secondary school	22.3%	16.5%	12.6%
Apprentice	13.5%	4.5%	19.4%
Tafe, College	27.8%	20.0%	20.1%
University undergraduate degree	18.6%	30.0%	23.0%
Postgraduate degree	9.2%	21.9%	12.0%
Other training courses	8.4%	7.1%	12.9%
Profession			
Administrative	2.8%	14.8%	3.3%
Managerial	20.1%	32.6%	23.8%
Professional/Technical	25.1%	28.1%	25.7%
Operator	18.8%	2.9%	16.0%
Technician or Trade/Maintainers	21.8%	3.9%	21.9%
Camps and catering	1.3%	1.3%	0.0%
Logistics and supply chain	2.4%	1.6%	3.3%
Other	7.6%	14.8%	6.1%

### FIFO worker sample (2018)

The responses from the FIFO worker sample were collected between November 2017 and February 2018. The 2018 FIFO worker sample (*N* = 3108) consisted mainly of men (82.8%) and had an average age of around 41 (*M* = 40.85; *SD =* 10.59) years of age. The most frequent highest levels of education were TAFE/college (27.8%), completion of secondary school (22.3%), and a university undergraduate degree (18.6%).

The 2018 FIFO worker sample was highly representative of the broader Western Australian resource sector population. With regard to industry according to the Australian Bureau of Statistics (Australian Bureau of Statistics, 2018a), proportions were similar in terms of mining (WA resources 94,400 people, 84%; FIFO sample 2577 people, 82.9%) versus oil and gas (WA resources 17,900 people, 19%; FIFO sample 531 people, 17.1%). The FIFO worker sample was also representative of WA resources sector workers (Australian Bureau of Statistics, 2018a) with respect to gender (WA resources male 81.6%, female 18.4%; FIFO sample male 82.8%, female 17.1%), and age (around 80% being from 25 to 54 years old).

### Benchmark sample (2018)

Responses from a benchmark sample (*N* = 326) of non-FIFO workers were collected through purposeful sampling. Using quotas on key demographics ensured that the benchmark samples, like the FIFO sample, consisted of males (77.3%) from Western Australia (78.0%) and matched the FIFO sample in terms of marital status. Despite our efforts, the benchmark sample differed from the FIFO worker sample in its age (*M* = 50.7 years; *SD* = 11.31), educational level (over 50% had completed a university undergraduate or postgraduate degree) and their professional roles (with a higher representation of administrative and managerial roles).

### FIFO worker sample COVID-19 (2020)

The 2020 FIFO sample included n = 362 FIFO workers. The data were collected between May and October 2020. The sample was mostly male (81.6%), and mostly aged between 35 and 54 years. Most participants reported being married or in a domestic partnership (75%), with at least one dependent child (59%). Participants reported to be mostly engaged in mining (61%), oil and gas (22%), and construction (8%) industries. The reported highest level of education completed was most commonly TAFE or traineeship (39.5%), and university undergraduate degree (23%).

### Additional comparison data used

In addition to the data collected from the samples listed above, a pre-existing data set from the 2017–2018 National Health Survey (Australian Bureau of Statistics, 2018b) was used as norm data for Kessler-10 data on psychological distress (depression and anxiety) in the wider Australian population before COVID-19. The 2017–2018 sample was nationally representative of the male Australian adult population (*N* = 8,658 completed the K10 measure). For the comparison with the FIFO worker samples (as a majority male workforce), male respondents were specifically included in the comparison to the population K10 mean using the mean sum scores of psychological distress (K10, anxiety and depression) for the 2018 FIFO sample and the existing data sets. We also include data on psychological distress reported by Rahman et al. (2020; *N* = 587) that was collected from the wider Australian population in 2020 to compare with the FIFO workers in 2020.

### Procedure

This study was approved by university ethics committees at the University of Western Australia and Curtin University. Ethics board approval number University of Western Australia: RA/4/1/9262; Ethics board approval number Curtin University: HRE2018–0449.

The 2018 FIFO worker responses were collected via an online and pen and paper survey. The survey was advertised via industry bodies, companies, unions, and mental health organisations. To generate the comparison sample that matched key attributes of the FIFO worker sample, a data collection company was engaged. The 2020 FIFO worker responses were collected by online survey, advertised through industry bodies, companies, and social media. In all surveys, participation was voluntary and confidential. Only high-quality responses were included in both samples, with cases retained if: multiple careless response checks were passed and there was a response time of at least 2 s per item (Ward & Meade, 2018) and at least 70% of the survey was completed (Dittman et al., 2016).

### Measures

*Psychological distress*, including feelings of depression, restlessness, fatigue, worthlessness, and anxiety, was measured via the Kessler-10 (K10; Kessler et al., 2002). Responses to items were on a 5-point scale (ranging from 1-None of the time, to 5-All of the time). The items were reliable with a Cronbach’s α = .92 or higher. A summed score across all items was computed.

*Wellbeing* was measured using a shortened version (nine items) of the Mental Health Continuum (Lamers et al., 2011) which assesses social wellbeing (i.e., social integration, contribution, coherence, actualisation and acceptance); emotional wellbeing (i.e., positive emotions); and psychological wellbeing (i.e., self-acceptance, growth, purpose). Respondents rated the frequency of every wellbeing aspect in the past month on a 6-point Likert scale (ranging from 1-Never to 6-Every day). We shortened the scale to reduce the burden on participants by selecting the highest loading items in each dimension. Cronbach’s alphas for each wellbeing dimension were high (Cronbach’s α = .81 or higher). Mean scores were computed for each subscale.

*Burnout* is a state of mental exhaustion due to prolonged exposure to work-related stressors (Taris et al., 1999). Two items from the Maslach Burnout Inventory exhaustion subscale were used (“I feel emotionally drained from my work”; and “I feel used up at the end of the work day”) as per Dollard and Bakker (2010). Responses were on a 7-point scale from 1-Never to 7-Every day. Mean scores were computed for the two items (Cronbach’s alpha was .87 and over).

*Suicide intention* was measured via three items from the Self-Injurious Thoughts and Behaviours Interview (Nock et al., 2007). The items ask about thoughts and plans about suicide on an 8-point agreement scale from 1-Strongly disagree to 8-Strongly agree (Cronbach’s α = .61). A mean score was computed.

*Control variables* included were age, gender, education, and professional role, given the variation between the FIFO workers and the benchmark sample on these demographics. Education was coded into two dummy coded variables: one for higher education (university undergraduate and postgraduate) and one for college (apprentice, TAFE, college, other training courses). The professional role was dummy coded to represent whether the worker was an operator/technician vs administrative, managerial and professional role.

### Analysis

First, we provide a comprehensive descriptive overview of the psychological distress scores for each of the samples and data sets reported on, by clustering participants into groups that represent low, moderate, high, and very high psychological distress scores following guidelines by Andrews and Slade (2001). Second, we compare the 2018 FIFO worker sample with the benchmark sample from 2018, and the 2018 FIFO worker sample with the 2020 FIFO worker sample using non-parametric statistical analyses. In each of these comparisons the sample sizes were unequal, the data and its residuals were not normally distributed, and the variances were not equal between samples. Accordingly, we used Welch’s t-tests to compare the means of the 2018 FIFO worker sample with the 2018 benchmark sample, and then the 2018 FIFO worker sample with the 2020 FIFO worker sample. This approach allowed us to document the impact of FIFO work per se. We further conduct hierarchical regression analyses with bootstrapping (1000 bootstrap samples, 95% confidence intervals; see Hayes, 2017); entering control variables in step 1 and a dummy coded FIFO work variable (FIFO work = 1; benchmark sample = 0) in step 2 for the 2018 FIFO worker sample with the 2018 benchmark sample. The regression analysis provides insights into the extent to which FIFO vs other work explain additional variance beyond demographic characteristics (age, gender, education, and professional role) in the mental health and wellbeing outcomes (a dummy coded variable was also used for comparing scores from 2018 and 2020). Third, we performed a one sample t-test to compare the FIFO worker sample from 2018 means to the Australian norm group means from the Australian Bureau of Statistics (Australian Bureau of Statistics, 2018b, 2019). An overview of correlations is provided in the Appendix.

## Results

Table 2 provides an overview of the K10 scores, respectively, for each of the samples and existing data sets that were included in this study (please see Appendix for an overview of correlations).Psychological distress scores (K10) given via percentages representing different categories of psychological distress for 2018 samples show that 21.8% and 10.8% of the FIFO worker sample had high and very high psychological distress, respectively, compared to approximately 8.0% and 3.7% of the Australian norm sample and 12.7% and 4.6% of the benchmark group before COVID-19. A total of 68% of the Australian norm group and 55.84% of the matched benchmark sample reported low psychological distress, whereas only 37.1% of the FIFO workers included in this study reported low levels of psychological distress. The 2020 FIFO worker sample recorded an increased number of workers experiencing high or very high levels of psychological distress (40.9%) as well as fewer FIFO workers experiencing low levels of psychological distress (27.4%). In data collected from the wider Australian population (Rahman et al., 2020) fewer people fell into the high to very high psychological distress category (37.3%) and a larger group responded with low levels of psychological distress compared to the FIFO workers (37.5%).

**Table 2.** Overview of FIFO workers and comparison samples from 2020 to 2018 with levels of psychological distress (K10).

	2020 COVID FIFO sample % (K10)	2018 FIFO sample % (K10)	2018 matched benchmark group % (K10)	2018 Australian men norm data % (K10)	2020 COVID Australian sample %(Rahman et al., 2020; K10)
Low	27.4	37.1	55.84	65.9	37.5
Moderate	31.7	30.3	26.95	22.1	29.3
High	28.3	21.8	12.66	8.4	20.3
Very High	12.7	10.8	4.55	3.6	13.0
High + Very high combined	40.9	32.6	17.21	12.0	33.3

To investigate the difference in mental health of FIFO workers and other workers before COVID-19, FIFO workers (2018 sample) were first compared to the 2018 benchmark sample. An overview of the state of mental health in FIFO workers and the benchmark sample is provided in Table 2. Results of the Welch’s test indicate significant differences in all mental health aspects between FIFO workers and the benchmark sample (see Table 3). Consistent across these mental health aspects, FIFO workers were shown to have worse mental health and wellbeing than the benchmark group. Further, the statistical comparison using regression analyses (see Table 4) showed significant differences persisting for two of the mental health indicators after controlling for demographic characteristics in 2018. First, for psychological distress, when added in Step 2 following the demographic variables, FIFO work accounted for an additional 0.3% of the variance in psychological distress, with unstandardised regression coefficient B = 1.440 (95% CI .574, 2.206), p = .001.Second, FIFO work added in step 2 accounted for an additional 1% of the variance in burnout, with unstandardised regression coefficient B = .654 (95% CI .436, .867), p = .001. While the amount of additionally explained variance was small, FIFO work significant explained psychological distress and burnout over and above demographic variables. No statistically significant differences for wellbeing or suicidal thoughts were indicated after controlling for demographics (see Table 5).

**Table 3.** Comparison of mental health and wellbeing between FIFO and benchmark samples (2018).

				*Welch’s t-test*			
Construct	Group	*M*	*SD*	*df*		*F*	*p*
Psychological distress (K10)	FIFO	19.36	7.14	Between	1	68.25	.000
	Benchmark	16.30	6.07	Within	398.18		
Burnout	FIFO	3.88	1.33	Between	1	73.58	.000
	Benchmark	3.00	1.72	Within	372.49		
Emotional wellbeing	FIFO	4.47	1.12	Between	1	8.48	.004
	Benchmark	4.65	1.09	Within	375.98		
Social wellbeing	FIFO	3.38	1.33	Between	1	24.24	.000
	Benchmark	3.74	1.24	Within	381.78		
Psychological wellbeing	FIFO	4.17	1.19	Between	1	8.77	.003
	Benchmark	4.35	1.03	Within	395.27		
Suicide intention	FIFO	1.77	1.37	Between	1	6.22	.013
	Benchmark	1.57	1.23	Within	325.81

**Table 4.** Regression analyses using the 2018 FIFO worker sample and the benchmark sample from 2018.

	K10		Burnout		Emotional WB		Social WB		Psychological WB		Suicide intention	
	*B*	95% CI	*B*	95% CI	*B*	95% CI	*B*	95% CI	*B*	95% CI	*B*	95% CI
Step 1												
Age	−.13**	−.15, −.11	−.03**	−.03, −.02	.01**	.00, .01	.01**	.01, .01	.01*	.00, .01	−.01*	−.01, −.00
Gender	−.44	−1.13, 1.13	−.19*	−.34, −.02	−.13*	−.24, −.02	−.15*	−.27, −.03	−.07	−.17, .05	.07	−.07, .20
Prof role	−.94*	−1.51, −.41	−.04	−.17, .09	.09	−.00, .18	.22**	.12, .32	.17*	.08, .26	−.05	−.18, .07
Education: TAFE	−.37	−1.07, .27	.01	−.15, .16	.05	−.05, .16	.03	−.09, .14	.03	−.08, .14	−.13	−.26, .03
Education: university	−1.70**	−2.49, −.91	−.21*	−.40, −.02	.17*	.05, .31	.35**	.22, .48	.18*	.04, .30	−.32*	−.18, .07
R^2^	.05		.03		.01		.04		.02		.01	
Step 2												
Age	−.12**	−.14, −.09	−.02**	−.03, −.02	.01*	.00, .01	.01**	.00, .01	.00*	.00, .01	−.01*	−.01, −.00
Gender	−.48	−1.16, .20	−.21*	−.36, −.05	−.13*	−.24, −.02	−.15*	−.27, −.03	−.06	−.17, .05	.07	−.07, .20
Prof role	−.86	−1.41, −.34	−.01	−.14, .13	.08	−.01, .17	.21**	.11, .31	.16*	.07, .25	−.05	−.17, .07
Education: TAFE	−.38*	−1.09, .27	.00	−.15, .15	.05	−.05, .16	.03	−.09, .14	.03	−.08, .14	−.13	−.26, .03
Education: university	−1.58**	−2.36, −.80	−.16	−.35, .03	.17*	.04, .30	.34**	.20, .47	.17*	.04, .30	−.31*	−.48, −.14
FIFO work	1.44**	.57, 2.21	.65**	.44, .87	−.08	−.21, .05	−.16	−.31, .01	−.06	−.19, .06	.08	−.09, .24
R^2^	.06		.04		.01		.04		.02		.01	
∆R^2^	.00		.01		.00		.00		.00		.00	

Note: CI=confidence intervals, **p ≤ .01, *p < .05; FIFO variable coded 1 = FIFO, 0 = Not FIFO; gender coded 0=female 1 = male; prof role coded 1 = managerial and professional roles, 0 = frontline roles (e.g., operators, administrators, drivers, cleaners, traders, caterers)s.

**Table 5.** Comparison of FIFO worker samples and Australian population norm psychological distress (before COVID-19, data from 2018).

				One sample t-test			
	Group	*M*	*SD/SE*	df		T	p-value
Before COVID-19 (data from 2018)							
K10	FIFO	19.36	SD = 7.14	Between	1		
	Norm	15.50	SE^1^ = 0.13	Within	3041	29.77	<.000
K10 Men	FIFO	19.25	SD = 7.12	Between	1		
	Norm	15.10	SE = 0.14	Within	2521	29.26	<.000
K10 Women	FIFO	19.91	SD = 7.27	Between	1		
	Norm	15.90	SE = 0.10	Within	515	12.54	<.000

Next, one sample t-tests comparing the FIFO worker sample with the average score of the Australian norm data (both from 2018) showed that the scores for the FIFO sample on psychological distress were significantly higher than for the norm group (*t* (3041) = 29.77 *p* = .000) before COVID-19 (see Table 4).

To address our second aim of identifying the impact of COVID-19 on FIFO worker mental health, we compare FIFO worker mental health reported in 2018 with scores from 2020. First, results from Welch’s t-tests reported in Table 6 indicate a significantly higher level in psychological distress (*F*(1, 286.65) = 9.04; *p* = .003) as well as significantly lower emotional wellbeing (*F*(1, 289.55) = 12.21; *p* < .000) in 2020 compared to 2018. After controlling for demographic variables in hierarchical regression analyses (see Table 7), the differences between FIFO workers in 2018 and 2020 remained significant for psychological distress B = 1.07 (95% CI 0.55, 1.56), p = .001, and emotional wellbeing B = −0.17 (95% CI −.25, −.08), p = .001. Additionally, after controlling for demographics significantly higher suicidal intention was indicated in 2020 compared to 2018 in FIFO workers B = 0.16 (95% CI .01, .23), p = .05. No difference was found for social or psychological wellbeing, across the two time points. Comparing FIFO workers’ levels of psychological distress to a sample pulled from the wider Australian population during COVID-19 using a one sample t-test showed FIFO workers had worse mental health (*t* (250) = 2.61, *p* = .01).

**Table 6.** Comparison of mental health and wellbeing between FIFO worker samples in 2018 (before COVID-19) and 2020 (during COVID-19).

				*Welch’s t-test*			
	Group	*M*	*SD*	*df*		*F*	*p*
Before and during COVID-19 (2018 & 2020)							
Psychological distress (K10)	FIFO 2018	19.36	7.20	Between	1	9.04	.003
	FIFO 2020	20.87	4.8	Within	286.65		
Social Wellbeing	FIFO 2018	3.38	1.33	Between	1	.026	.873
	FIFO 2020	3.35	1.27	Within	300.37		
Emotional Wellbeing	FIFO 2018	4.47	1.12	Between	1	12.21	<.001
	FIFO 2020	4.18	1.21	Within	289.55		
Psychological Wellbeing	FIFO 2018	4.17	1.19	Between	1	2.28	.132
	FIFO 2020	4.06	1.12	Within	293.11		
Suicide intention	FIFO 2018				1	3.55	.061
	FIFO 2020				276.87

**Table 7.** Regression analyses using the 2018 and 2020 FIFO worker samples.

	K10		Emotional WB		Social WB		Psychological WB		Suicide intention	
	*B*	95% CI	*B*	95% CI	*B*	95% CI	*B*	95% CI	*B*	95% CI
Step 1										
Age	−.10**	−.13/-.08	.004	.00/.01	.01**	.00/.01	.00	.00/.01	−.01	−.01/.00
Gender	−.46	−1.13/.19	−.12*	−.22/-.01	−.19*	−.31/-.06	−.07	−.18/.04	.05	−.10/.19
Prof role	−.87**	−1.45/-.27	.08	−.02/.17	.23**	.13/.33	.17**	.07/.28	−.03	−.16/.10
Education: TAFE	−.35	−.99/.29	.02	−.07/.14	.04	−.07/.16	.05	−.06/.16	−.13	−.28/.02
Education: university	−1.56**	−2.35/-.77	.18*	.05/.31	.35**	.20/.49	.20*	.07/.35	−.33**	−.52/-.14
R^2^	.035**		.010**		.033**		.015**		.009**	
Step 2										
Age	−.11**	−.13/-.09	.01*	.00/.01	.01**	.00/.01	.00	.00/.01	−.01*	−.01/.00
Gender	−.42	−1.12/.23	−.12*	−.23/-.02	−.19*	−.31/-.06	−.07	−.19/.03	.05	−.09/.19
Prof role	−.84*	−1.43/-.26	.08	−.02/.17	.23**	.13/.33	.17**	.07/.28	−.02	−.15/.10
Education: TAFE	−.44	−1.10/.19	.04	−.06/.15	.05	−.07/.17	.05	−.05/.16	−.14	−.29/.01
Education: university	−1.69**	−2.49/-.92	.20*	.08/.33	.35**	.21/.50	.22*	.08/.36	−.35**	−.53/-.15
Year	1.07**	.55/1.56	−.17**	−.25/-.08	−.04	−.12/.05	−.09*	−.17/.00	.16*	.01/.23
R^2^	.040**		.016**		.033		.016		.011*	
∆R^2^	.005		.006		.000		.001		.002	

Note: CI=confidence intervals, **p ≤ .01, *p < .05; gender coded 0 = female 1 = male; prof role coded 1 = managerial and professional roles, 0 = frontline roles (e.g., operators, administrators, drivers, cleaners, traders, caterers); Year coded 0 = 2018 1 = 2020.

## Discussion

The results of this study provide insights into the subjective mental health and wellbeing of Australian-based FIFO workers before and during COVID-19. The study captured the wide spectrum of mental health ranging from mental-ill health and suicidal thoughts to mental health and wellbeing (Greenspoon & Saklofske, 2001; World Health Organisation, 2013).

We identify three key insights from this study. First, the results show that FIFO workers are at a greater risk of mental ill-health (i.e., depression, anxiety, burnout, and suicide intention) and lower wellbeing in comparison to other groups. Second, our results identify that the worse mental health of FIFO workers compared to other workers was only partially attributable to their demographics. Differences in mental health persisted for some mental ill-health measures (i.e., psychological distress and burnout) when demographic variables were controlled for. Overall, this illustrates that FIFO workers are an at-risk group. They are so only in part because of their age, gender, level of education, and professional roles, all of which have been previously noted as risk factors (Education and Health Standing Committee, 2015), and in part because of the nature of the FIFO experience itself (before and during COVID-19). Third, the results show that mental health of FIFO workers was worse during COVID-19 compared to before (i.e., regarding psychological distress, emotional well-being and suicidal intention) and that their mental health was worse than that of the wider population. Overall, these results illustrate the nuanced nature in which FIFO work may be affecting workers, a consideration that remains relevant, and is in fact amplified in its relevance during COVID-19. The findings of this study can move the debate and research literature around FIFO worker mental health beyond being stuck at deciding “if” and move the conversation towards the “why” and how to change things for the better.

Our results demonstrate that mental health outcomes for workers are accounted for by being engaged in FIFO work beyond demographics on many mental health outcomes. Yet, they also show that demographics are relevant to understanding FIFO workers' mental health. We identify that the demographic make-up of the FIFO workforce is not easily changed, for example, through selective hiring practices and doing so may in fact not be a feasible or desirable, due to enduring masculine work culture and other issues (Laplonge, 2016). So, a conclusion to that end may be limited in its utility, if not counterproductive. Instead, we suggest that it will be more beneficial to recognise that FIFO workers, as they are in terms of demographic makeup and work conditions, are an at-risk group for mental health and to direct our focus onto the specific aspects of FIFO work that could be changed to protect FIFO workers. Thus, we propose to focus on protective factors such as boosting support, managing work design (including workload), and humanising work culture by considering justice systems at work, and supporting social connection (Gilbert, 2019). In addition, the perceived stigma to seeking mental health services in case of mental health issues (Tynan et al., 2016) needs to be considered given our study’s findings of FIFO workers’ heightened levels of mental-ill health and lower levels of wellbeing.

Some methodological issues need to be considered when interpreting our findings. First, the results show a static picture of mental health and wellbeing in FIFO workers and others. Research grounded in the stability and change model byOrmel and Schaufeli (1991) shows that around 50%–60% of variation in psychological distress is the result of a stable factor, with the remainder being subject to fluctuation based on state level psychological distress, which is likely affected by life events and other temporal aspects (Breslin et al., 2006). Our cross-sectional data cannot account for such fluctuations. Second, the results indicate differences between FIFO workers across years and compared to other groups, allowing some conclusion of causality. However, some limitations in asserting causality need to be recognised. For example, it is unclear whether at-risk individuals self-select into FIFO work arrangements. Further, there were indeed some differences in the demographics between the three samples included in this study. Key to our findings is that we controlled for such differences in our analyses. Finally, other factors were not controlled for in our study like differences in weather or economic changes. Irrespective of these issues, it needs to be recognised that the differences that are reported in our study raise an issue that warrants attention and identifies FIFO workers as an at-risk group for mental ill-health and wellbeing

In conclusion, our study shows a refined picture of the extent to which FIFO workers are an at-risk group in terms of their mental health. It further shows that this is even more so the case since COVID-19. The results illustrate the importance for FIFO workers and employers to pay attention to mental health. Industry, government, and other relevant stakeholders should ensure that the support options they can provide suit the constraints of FIFO work. Given the higher levels of mental-ill health and poorer wellbeing in FIFO workers during COVID-19 offering support and designing work to address these issues is now even more important.

## Data Availability

The data are not publicly available due to their containing information that could compromise the privacy of research participants.

## Appendix

**Table:**

	1	2	3	4	5	6	7	8	9	10
**2018 FIFO worker sample**										
1. Age										
2. Gender	.17**									
3. Professional Role	.14**	.11**								
4. Education: TAFE	.18**	.17**	−.07**							
5. Education: university	−.18**	−.17**	.07**	−1.00**						
6. K10	−.14**	−.04	−.08**	.09**	−.09**					
7. Burnout	−.11**	−.07**	−.01	.03	−.03	.65**				
8. Emotional wellbeing	.02	−.05**	.05*	−.08**	.08**	−.66**	−.48**			
9. Social wellbeing	.03	−.07**	.08**	−.15**	.15**	−.48**	−.40**	.61**		
10. Psychological wellbeing	.01	−.04*	.08**	−.10**	.10**	−.58**	−.41**	.73**	.61**	
11. Suicide intention	−.02	.02	−.03	.09**	−.09**	.35**	.19**	−.32**	−.19**	−.28**
**2018 Benchmark sample**										
1. Age										
2. Gender	.19**									
3. Professional Role	.06	.12*								
4. Education: TAFE	.14*	.05	−.10							
5. Education: university	−.14*	−.05	.10	−1.00**						
6. K10	−.35**	−0.10	.01	−.05	.05					
7. Burnout	−.28**	.00	.19**	−.11*	.11*	.61**				
8. Emotional wellbeing	.20**	.05	.03	.11	−.10	−.62**	−.38**			
9. Social wellbeing	.12*	0.09	.07	.00	−.00	−.37**	−.28**	.58**		
10. Psychological wellbeing	.18**	.06	.14*	.04	−.04	−.50**	−.30**	.73**	.58**	
11. Suicide intention	−.06	.06	.00	−.05	.05	.25**	.14*	−.32**	−.15*	−.29**
**2020 FIFO worker sample**										
1. Age										
2. Gender	.15*									
3. Professional Role	.16*	.05								
4. Education: TAFE	.14*	.26**	.01							
5. Education: university	−.14*	−.26**	−.01	−1.00**						
6. K10	−.16	−.06	−.11	.00	.00					
8. Emotional wellbeing	.09	0.1	.07	−.05	.05	−.72**				
9. Social wellbeing	.12	.05	.12	−.03	.03	−.49**		.59**		
10. Social wellbeing	.01	.07	.16*	−.14*	.14*	−.67**		.72**	.61**	
11. Suicide intention	−.05	.01	−.17**	−.03	.03	.25**		−.24**	−.13*	−.31**

Note: **p ≤ .01, *p < .05; gender coded 0 = female 1 = male; prof role coded 1 = managerial and professional roles, 0 = frontline roles (e.g., operators, administrators, drivers, cleaners, traders, caterers).
